# Novel natural and synthetic inhibitors of solute carriers SGLT1 and SGLT2

**DOI:** 10.1002/prp2.504

**Published:** 2019-07-30

**Authors:** Paul Oranje, Robin Gouka, Lindsey Burggraaff, Mario Vermeer, Clément Chalet, Guus Duchateau, Pieter van der Pijl, Marian Geldof, Niels de Roo, Fenja Clauwaert, Toon Vanpaeschen, Johan Nicolaï, Tom de Bruyn, Pieter Annaert, Adriaan P. IJzerman, Gerard J. P. van Westen

**Affiliations:** ^1^ Unilever Research & Development Vlaardingen The Netherlands; ^2^ Division of Drug Discovery & Safety, Leiden Academic Centre for Drug Research Leiden University Leiden The Netherlands; ^3^ Drug Delivery and Disposition, Department of Pharmaceutical and Pharmacological Sciences KU Leuven Leuven Belgium

**Keywords:** diabetes, glucose, inhibitors, screening, SGLT1, SGLT2

## Abstract

Selective analogs of the natural glycoside phloridzin are marketed drugs that reduce hyperglycemia in diabetes by inhibiting the active sodium glucose cotransporter SGLT2 in the kidneys. In addition, intestinal SGLT1 is now recognized as a target for glycemic control. To expand available type 2 diabetes remedies, we aimed to find novel SGLT1 inhibitors beyond the chemical space of glycosides. We screened a bioactive compound library for SGLT1 inhibitors and tested primary hits and additional structurally similar molecules on SGLT1 and SGLT2 (SGLT1/2). Novel SGLT1/2 inhibitors were discovered in separate chemical clusters of natural and synthetic compounds. These have IC_50_‐values in the 10‐100 μmol/L range. The most potent identified novel inhibitors from different chemical clusters are (SGLT1‐IC_50_ Mean ± SD, SGLT2‐IC_50_ Mean ± SD): (+)‐pteryxin (12 ± 2 μmol/L, 9 ± 4 μmol/L), (+)‐ε‐viniferin (58 ± 18 μmol/L, 110 μmol/L), quinidine (62 μmol/L, 56 μmol/L), cloperastine (9 ± 3 μmol/L, 9 ± 7 μmol/L), bepridil (10 ± 5 μmol/L, 14 ± 12 μmol/L), trihexyphenidyl (12 ± 1 μmol/L, 20 ± 13 μmol/L) and bupivacaine (23 ± 14 μmol/L, 43 ± 29 μmol/L). The discovered natural inhibitors may be further investigated as new potential (prophylactic) agents for controlling dietary glucose uptake. The new diverse structure activity data can provide a starting point for the optimization of novel SGLT1/2 inhibitors and support the development of virtual SGLT1/2 inhibitor screening models.

**Abbreviations**
1‐NBD‐glucose: 1‐N‐(7‐nitrobenz‐2‐oxa‐1,3‐diazol‐4‐yl)amino)‐1‐deoxyglucose
APC: angular pyranocoumarin
BSA: bovine serum albumin
CHO: Chinese hamster ovary
DMEM: Dulbecco's Modified Eagle's Medium
DMSO: Dimethyl sulfoxide
FCFP6: functional connectivity fingerprint of 6 atoms
HBSS: Hanks' Balanced Salt Solution
HI‐FBS: heat inactivated fetal bovine serum
hSGLT: human sodium glucose linked transporter
NEAA: nonessential amino acids
*P p*.‐extract: *Peucedanum praeruptorum* extract
PC: principal components
PCA: principal components analysis/analyses
PCM: proteochemometric
PMD: physicochemical molecular descriptors
SD: standard deviation
SGLT: sodium glucose linked transporter
SGLT1/2: SGLT1 and/or SGLT2
SGLT2/SGLT1‐_50__μmol/L_: ratio of SGLT2 to SGLT1 activities with 50 μmol/L inhibitor
SLC: solute carrier
S_xc_: similarity of compound x to the central cluster compound c
SMILES: simplified molecular-input line-entry specification
T1DM: type 1 diabetes mellitus
T2DM: type 2 diabetes mellitus
TEER: transepithelial electrical resistance
TMS: Tetramethylsilane
VC: vehicle control

## 1 INTRODUCTION

Type 2 Diabetes Mellitus (T2DM) is a metabolic disease characterized by prolonged hyperglycemia. In healthy individuals, insulin signaling controls blood glucose levels by inducing tissues to absorb glucose from the circulation. In T2DM, peripheral tissues and the liver gradually become insensitive to insulin. The resulting hyperglycemia can induce a range of pathologies, in particular cardiovascular disease. T2DM is a growing pandemic,^1^ and there is a need for alternatives to insulin therapy. Powerful glucose–lowering drugs are available for T2DM.^2^ However, these can cause hypoglycemia resulting in considerable morbidity and mortality.^3^ Glycemic control therapies should therefore prevent both hyperglycemia and hypoglycemia. Accordingly, inhibitors of the Sodium Glucose Linked Transporters type 1 and 2 (SGLT1 and SGLT2) have emerged as insulin independent antihyperglycemic remedies.^4^, ^5^, ^6^, ^7^

SGLTs are members of the superfamily of Solute Carrier transporter proteins (SLC) and are cotransporters of monosaccharides that rely on the concentration gradient of sodium ions to transport molecules across cell membranes. SGLT1 is a high‐affinity, low‐capacity transporter that absorbs D‐glucose in the small intestine. SGLT2 has a lower affinity, but higher capacity for D‐glucose and is expressed in the kidney proximal glomerular tubules where it reabsorbs ≥90% of glucose from the primary urine.^2^, ^8^ The natural glucosylated‐dihydrochalcone phloridzin is a recognized SGLT inhibitor,^9^, ^10^, ^11^ and its glucosuric effect has long been known.^12^ Phloridzin was shown to correct hyperglycemia in rats.^13^, ^14^ Oral intake of phloridzin containing apple extracts caused blood glucose lowering and glucosuria in animal and human studies, but phloridzin was not detected in plasma or urine and the glucuronide metabolites of its aglycon phloretin positively correlated with glucosuria.^15^, ^16^ Thus, the hypoglycemic action of phloridzin involves metabolic and systemic effects that are not entirely understood. Furthermore, phloretin was shown to have undesirable bioactivities in vitro, including inhibition of various transporters, inhibition of oxidative phosphorylation and estrogenic activity.^17^

SGLT2 selective, rapidly absorbed phloridzin analogs were synthesized to prevent discomfort from high colonic glucose due to intestinal SGLT1 inhibition.^18^, ^19^, ^20^ These gliflozins efficaciously and safely reduced hyperglycemia in humans.^21^ While the glucosuria resulting from taking these drugs prevented weight gain or even induced weight loss in T2DM‐patients,^6^ it can lead to urinary tract infections.^21^ Moreover, SGLT2 inhibitors were shown to have a lower efficacy in individuals with impaired renal function.^22^ As 40% of T2DM‐patients have some degree of nephropathy, these drugs could have a reduced efficacy in individuals who would benefit the most.^7^ Alternatively targeting intestinal SGLT1 to inhibit glucose absorption has therefore gained considerable interest.^4^, ^23^, ^24^ The dual SGLT1/SGLT2 inhibitor sotagliflozin has shown efficacious and safe glycemic control in T2DM patients and healthy individuals.^25^, ^26^, ^27^ Furthermore, sotagliflozin increased plasma levels of the incretin GLP‐1 and the anorexigenic PYY.^28^ Recently, a nonabsorbable sotagliflozin analog was developed, LX2761, that acts as a selective SGLT1 inhibitor^29^ and improved glycemic control associated with increased circulating GLP‐1 in rodents.^30^

A natural, moderately active SGLT1 blocker causing no gastrointestinal discomfort would offer a good prophylactic for (pre‐) diabetic individuals.^31^ This study aimed to identify molecules outside the chemical space of phloridzin and structurally similar glycosides with moderate SGLT1 inhibitory activity. We screened a bioactive compound library, using an in vitro SGLT1 inhibition assay with a fluorescent glucose derivative as substrate.^32^ Screening hits were explored by in vitro (re)testing of these and additional structurally similar compounds, on SGLT1 and SGLT2 (SGLT1/2). We identified various structurally diverse, novel natural and synthetic inhibitors of SGLT1/2 in distinct chemical clusters. The dataset of diverse SGLT1/2 inhibitors with varying activity was used to develop an in silico proteochemometric (PCM) SGLT1 screening model.^33^ Finally, the activity of an identified novel natural inhibitor and a plant extract containing this compound were tested in a more physiologically relevant setting with SGLT1–expressing Caco‐2 cells,^34^ and the close glucose analog ^14^C‐α‐methylglucose as substrate. This study provides starting points for the development and optimization of novel SGLT1/2 inhibitors.

## 2 MATERIALS AND METHODS

### 2.1 Materials

Dulbecco's Modified Eagle's Medium (DMEM)‐F12, Heat Inactivated Fetal Bovine Serum (HI‐FBS) (both Biowest), Hanks' Balanced Salt Solution (HBSS) without Ca and Mg, Dulbecco's Phosphate Buffered Saline (both HyClone), Isopropanol, Methylene chloride (both Merck), 25% NaOH (Alfa Aesar), Ultima Gold Scintillation cocktail (Perkin Elmer), polypropylene 96‐well plates (Nunc), DMEM, ToxiLight bioassay kit (both Lonza), 15 cm cell culture dishes (Corning), clear‐bottom black 96‐well plates and white 96‐well Cellstar plates (both Greiner Bio‐One) were all obtained from VWR (Amsterdam, The Netherlands). TrypLE Express, Geneticin, Opti‐MEM, D‐glucose free DMEM, 5000 U/mL Penicillin‐Streptomycin, 100x MEM NonEssential Amino Acids (NEAA) Solution (all Gibco), Lipofectamine 2000 and water‐soluble Probenecid (both Invitrogen), were all ordered from Thermo Fisher Scientific (Breda, The Netherlands). 1‐N‐(7‐Nitrobenz‐2‐oxa‐1,3‐diazol‐4‐yl)amino)‐1‐deoxyglucose (1‐NBD‐glucose) was custom synthesized by Mercachem (Nijmegen, The Netherlands). Bovine Serum Albumin (BSA), Poly‐L‐lysine hydrobromide mol. wt. 30 000‐70 000, cell culture grade Dimethyl sulfoxide (DMSO) and 24‐well transwell plates (Costar) were all acquired from Sigma‐Aldrich Chemie (Zwijndrecht, The Netherlands). ^14^C‐α‐methylglucose was acquired from Perkin Elmer (Groningen, The Netherlands). The human SGLT1 (hSGLT1) cDNA cloned in the pCMV6‐neo vector was purchased from Origene Technologies (Rockville, USA). The human SGLT2 (hSGLT2) cDNA was custom synthesized and cloned into the pcDNA3.1 vector by Thermo Fisher Scientific (Breda, The Netherlands). *Peucedanum praeruptorum* extract powder was obtained from Guangzhou Phytochem Sciences (Guangzhou, China). Deuterated chloroform containing 0.03% (v/v) Tetramethylsilane (TMS) was acquired from Eurisotop (Saint‐Aubin, France).

### 2.2 Compounds

The Spectrum Collection bioactive compound library was obtained from Microsource (Gaylordsville, USA) and its compounds with simplified molecular‐input line‐entry specification (SMILES) are shown in Table S1. The additional compounds with their SMILES and suppliers are listed in Table S2. Stock solutions of 100 mmol/L were prepared in DMSO and stored at −20°C.

### 2.3 Cell culture

Chinese Hamster Ovary K1 wild type cells (CHO‐wild type) were obtained from LGC Standards (Cat. No. ATCC CCL‐61, Wesel, Germany) and were maintained in DMEM‐F12 supplemented with 10% Heat Inactivated Fetal Bovine Serum (HI‐FBS) and split twice weekly. CHO‐hSGLT1/2 cells (passage numbers 10‐65) were maintained in DMEM‐F12 supplemented with 10% HI‐FBS and 400 μg/mL geneticin to select for cell clones containing the hSGLT1 and hSGLT2 vectors with a neomycin resistance gene. Caco‐2 cells were purchased from DSMZ (Cat. No. ACC 169, Lot 16, Braunschweig, Germany) and were maintained in DMEM supplemented with 20% HI‐FBS, 1X NEAA and 83 U/mL Penicillin‐Streptomycin and split twice weekly. All cells were maintained and incubated in a humidified incubator at 37°C with 5% CO_2_.

### 2.4 Generation of stable CHO‐hSGLT1 and CHO‐hSGLT2 cell lines

For generation of CHO cell lines stably expressing the hSGLT1 (SLC5A1) gene, or hSGLT2 (SLC5A2) gene, CHO‐wild type cells were seeded in a 12‐well culture plate at 1 × 10^5^ cells/well, grown overnight and transfected with 10 μg plasmid DNA and 4 μL Lipofectamine 2000 in 200 μL OptiMEM. The next day, the cells were collected by TrypLE Express treatment and transferred to 15 cm cell culture dishes in several dilutions in DMEM‐F12 supplemented with 10% HI‐FBS and 400 μg/mL geneticin to select for stable clones containing the hSGLT1 and hSGLT2 vectors that also carry a neomycin resistance gene. Cells were grown until small clones were visible and medium with geneticin was changed regularly. For each cell line, twenty clones were randomly selected, transferred to 48‐well culture plates, propagated and tested for 1‐NBD‐glucose and ^14^C‐α‐methylglucose uptake (data not shown). Clones with the highest substrate uptake levels were used for SGLT1 and SGLT2 inhibition assays.

### 2.5 SGLT1 inhibition assay for screening of the Spectrum Collection compound library

Two days before the assay, CHO‐wild type and CHO‐hSGLT1 cells were seeded in maintenance medium at 25 000 cells/well in clear‐bottom black 96‐well cell culture plates. Before the assay, cells were washed 3 times with 150 μL/well D‐glucose free DMEM with 0.3% (w/v) BSA. Library compounds at 50 μmol/L (singlicate) prepared in D‐glucose free DMEM with 160 μmol/L 1‐NBD‐glucose and 0.3% (w/v) BSA and negative controls (triplicate) were added at 100 μL/well and placed in a humidified incubator at 37°C with 5% CO_2_ for 90 minutes. Next, cells were washed 3 times with DMEM with 0.3% (w/v) BSA (150 μL/well). Care was taken to remove all medium after the last wash. Finally, 1‐NBD‐glucose was extracted from the cells by adding 10 μL/well isopropanol and orbital shaking for 5 minutes at 600 rpm, followed by incubation in the dark for 30 minutes. Fluorescence was measured on a Tecan Infinite M200 Microplate Reader (Tecan, Männedorf, Switzerland) with excitation at 445 nm and emission at 525 nm. The amount of 1‐NBD‐glucose taken up by the CHO‐wild type or CHO‐hSGLT1 cells in the presence or absence of test compounds was interpolated from 1‐NBD‐glucose calibration standards. The background uptake by CHO‐wild type was subtracted from the uptake by CHO‐hSGLT1. Background corrected uptake with test compound was expressed as a percentage of corrected uptake without test compound. In total, 1956 compounds were screened for SGLT1 inhibiting activity. An activity threshold was arbitrarily set at ≤70% of 1‐NBD–glucose uptake (or >30% inhibition) compared to negative control.

### 2.6 SGLT1 and SGLT2 inhibition assays for validation and investigation of screening hits and structurally similar compounds

Two days before the assay, CHO‐hSGLT1 or CHO‐hSGLT2 cells were seeded in maintenance medium at 60 000 cells/well in clear‐bottom black 96‐well plates, precoated with 100 μg/mL poly‐L‐lysine. Cells were washed with 240 μL/well D‐glucose free DMEM. Dilutions of test compounds and controls prepared in D‐glucose free DMEM with 350 μmol/L 1‐NBD‐glucose, 0.3% (w/v) BSA and 2 mmol/L probenecid (to inhibit efflux of 1‐NBD‐glucose) were added at 90 μL/well in duplicate and placed in a humidified incubator at 37°C with 5% CO_2_ for 30 minutes. The assay incubation media were removed and stored at −80°C for subsequent cytotoxicity analysis. Then, the cells were washed once with ice‐cold DMEM‐F12 and once with ice‐cold HBSS, both at 240 μL/well. Care was taken to remove all medium after each wash. Finally, 1‐NBD‐glucose was extracted from the cells with 100 μL/well isopropanol for 10 minutes at 600 rpm using an orbital shaker. Fluorescence was measured using a FlexStation 3 Multi‐Mode Microplate Reader (Molecular Devices, San Jose, USA) with excitation at 445 nm, emission at 525 nm and cut off 515 nm. The uptake of 1‐NBD‐glucose was normalized to the dynamic range between maximal uptake (≤0.5% (v/v) DMSO vehicle control, VC) and background uptake (with 15, 50 or 100 μmol/L phloridzin, all ≥50× SGLT1/2‐IC_50_ to achieve complete inhibition). The mean normalized SGLT1/2 activity at 5 and 50 μmol/L was calculated from n biological replicates as indicated in Table S2. IC_50_‐values were determined from logarithmic dose response curves from 0.2 to 500 μmol/L for test compounds and from 1 to 100 μmol/L for phloridzin, using four parametric fitting with GraphPad Prism version 5.04 software (La Jolla, USA). For some compounds, cell detachment was observed at 500 μmol/L and corresponding low fluorescence values were excluded from dose response curve fitting. Inhibition curves were used if R^2^ ≥ 0.95. The mean IC_50_‐values were calculated from n biological replicates as indicated in Table 1. If applicable, statistical outliers were determined using Grubbs’ test (alpha = 0.05) and excluded for calculation of the mean ± SD normalized SGLT1/2 activity and mean ± SD IC_50_‐values.

**Table 1:** SGLT1/2‐IC_50_‐values of the most active SGLT1 inhibitor per chemical cluster

Compound	Cluster	Type	SGLT1‐IC_50_ (μmol/L)			SGLT2‐IC_50_ (μmol/L)		
			n	Mean	SD	n	Mean	SD
Phloridzin	Glycosides	Natural	25^+^	0.34	0.14	29^+^	0.16	0.1
(+)‐pteryxin	APC	Natural	5	12	2	4	9	4
(+)‐ε‐viniferin	Viniferin‐like	Natural	4	58	18	2	110, 110	n.a.
Quinidine	Quinidine‐like	Natural	2	51, 74	n.a.	2	72, 40	n.a.
Cloperastine	Diphenhydramine‐like	Synthetic	4	9	3	6	9	7
Bepridil	Trimipramine‐like	Synthetic	4	10	5	8	14	12
Trihexyphenidyl	Trihexyphenidyl‐like	Synthetic	3	12	1	7	20	13
Bupivacaine	Bupivacaine‐like	Synthetic	4	23	14	5	43	29

IC_50_‐values of the most active novel inhibitors from chemical clusters presented in Figure 2. Results are means with SD from biological replicates, or individual measurements. n.a. = not applicable. + = original dataset contained a statistical outlier that was excluded from calculation of the mean and SD.

### 2.7 Cytotoxicity assay

The cytotoxicity of representative SGLT1/2 inhibitors and compounds from the different chemical clusters was tested. Cytotoxicity was determined using the ToxiLight bioassay kit according to the supplier's instructions. This nondestructive assay measures leakage of the enzyme adenylate kinase (AK) from damaged cells into the CHO‐SGLT1/2 inhibition assay media, ie the degree of cytolysis. Briefly, 20 μL of CHO‐SGLT1/2 inhibition assay medium was added to 100 μL reconstituted AK detection reagent in white 96‐well Cellstar plates and incubated for 5 minutes at room temperature. Next, bioluminescence was measured using a FlexStation 3 Multi‐Mode Microplate Reader (Molecular Devices) by 1 second integrated reading. Cytotoxicity was expressed as the percentage of bioluminescence of the DMSO vehicle control which was set at 0%. The mean cytotoxicity was calculated from n biological replicates (combining CHO‐SGLT1 and CHO‐SGLT2 data) as indicated in Table S2. If applicable, statistical outliers were determined using Grubbs’ test (alpha = 0.05) and excluded for calculation of the mean ± SD. CHO‐SGLT1/2 inhibition assay medium was not stored for all assays and cytotoxicity of some compounds was not analyzed or analyzed for two biological replicates only. Mean values ≥20% were considered toxic (arbitrary threshold). Most compounds showed cytotoxicity values between −20% and 20% of vehicle control. Only quinine showed a mean cytotoxicity value above the 20% threshold, albeit with a high standard deviation (SD), 21 ± 38% (Table S2). Non SGLT1 inhibiting stilbenoids from the viniferin–like cluster, but not (+)‐ε‐viniferin or (−)‐ε‐viniferin, produced low negative cytotoxicity values ranging from −42% to −89%, as well as the flavonoid glycoside wistin with −51%.

### 2.8 Preparation of an APC containing Peucedanum root extract

Angular pyranocoumarins (APCs) were extracted from *Peucedanum praeruptorum* root extract powder with methylene chloride. Batches of 0.5 g powder were shaken with 1.5 mL methylene chloride in a 2 mL Eppendorf tube for 30 minutes in an Eppendorf Thermomixer F1.5 (Eppendorf, Kerkenbos, The Netherlands) at 500 rpm. Then, the samples were centrifuged for 10 minutes at 14 000 rpm and the supernatant was transferred to a glass tube. The pellet was extracted a second time as described above. All supernatant fractions were pooled into a single glass tube and methylene chloride was removed by evaporation under a gentle stream of air. The final pellet was dissolved in 150 μL DMSO. Each extraction resulted in less than 1% (w/w) of the starting amount, eg 2 g of *Peucedanum praeruptorum* root extract powder resulted in 15 mg of *Peucedanum praeruptorum* extract (*P p*.‐extract).

### 2.9 NMR sample preparation and data acquisition

Dried *P p*.‐extract and (+)‐pteryxin were dissolved in deuterated chloroform with 0.03% (v/v) TMS using an Eppendorf Thermomixer C at room temperature. Subsequently, the samples were centrifuged for 5 minutes at 17 000 g at room temperature and 650 µL of supernatant was transferred to a 5‐mm NMR tube for analysis. 1D ^1^H‐NMR spectra were recorded with a ZG pulse sequence using a Bruker Avance III HD 700 spectrometer, equipped with a 5‐mm BBI probe. The probe was tuned to detect ^1^H resonances at 700.13 MHz. The internal probe temperature was set to 298 K and 64 scans were collected in 64k data points with a relaxation delay of 1 second and a spectral width of 20 ppm. The data were processed in Topspin v3.5 pl 1 (Bruker BioSpin GmbH, Rheinstetten, Germany). An exponential window function was applied to the free induction decay with a line‐broadening factor of 0.15 Hz prior to the Fourier transformation. Manual phase and baseline correction was applied to all spectra. The spectra were referenced against the methyl signal of TMS (δ 0.0 ppm).

### 2.10 Analysis of cellular uptake and transcellular transport of ^14^C‐α‐methylglucose by Caco‐2 cell monolayers

Caco‐2 cells (passage numbers 7 and 13) were seeded (29,700 cells) onto polycarbonate Transwell^®^ inserts (6.5 mm diameter and 3.0 μm pores) and cultured for 21‐24 days to obtain differentiated polarized monolayers. Apical and basal medium was refreshed twice a week and the final medium refresh was done the day before the uptake and transport experiment. TransEpithelial Electrical Resistance (TEER) was measured with an EVOM^2^ epithelial voltmeter (World Precision Instruments, Sarasota, USA) and inserts were used if TEER > 3000 Ω/cm^2^. First, cells were washed twice apically and basally with D‐glucose free DMEM. Then, 100 μL (+)‐pteryxin dilutions (50 μmol/L and 500 μmol/L), *P p*.‐extract dilutions (1/200 and 1/2000), or 0.5% (v/v) DMSO vehicle control were applied apical in D‐glucose free DMEM with 0.3% (w/v) BSA and 40 nCi/mL ^14^C‐α‐methylglucose. The basal acceptor wells (24‐well) contained 350 μL D‐glucose free DMEM with 0.3% (w/v) BSA. Then, cells were incubated in a humidified incubator at 37°C with 5% CO_2_ for 90 minutes. Inserts were transferred to a clean 24‐well plate and washed once with DMEM (with D‐glucose) and once with HBSS, with 1 mL at the basal and 100 μL at the apical side. Cells were lysed with 100 μL of 0.2 mol/L NaOH for 30 minutes at 37°C and lysates were analyzed by liquid scintillation counting on a Tri‐Carb 2910 TR (Perkin Elmer, Groningen, The Netherlands) to determine cellular ^14^C‐α‐methylglucose uptake. Basal media were analyzed accordingly to determine transcellular ^14^C‐α‐methylglucose transport. Mean ± SD ^14^C‐α‐methylglucose uptake and transport were calculated from two biological replicates, each consisting of four technical replicates.

### 2.11 Selection and clustering of SGLT1 screening hits and structurally similar compounds

From the primary Spectrum Collection SGLT1 screening, 30 hits were selected based on commercial availability. Molecules structurally similar to primary hits were identified using public online compound databases (chemspider.com, pubchem.ncbi.nlm.nih.gov/ Pubchem, mcule.com) and 101 additional compounds were selected based on commercial availability. All 131 compounds were clustered using a Dice fingerprint distance function,^35^ on predetermined molecular Functional Connectivity Fingerprint of 6 atoms (FCFP6). The cluster selection method was set on maximum dissimilarity and clusters were recentered to minimize the average distance within a cluster. The maximum distance between cluster centers was set at 0.6. Calculation of FCFP6 values and clustering was done using Pipeline Pilot software v9.2 (BIOVIA, San Diego, USA). Similarity of cluster compounds (x) was expressed as a percentage of the cluster center (c) which was set at 100% S_xc_ (Table S2).

### 2.12 Principal component analysis of compound clusters and Spectrum Collection compound library

Principal Component Analysis (PCA) of the 131 primary hits plus selected additional similar compounds and of the Spectrum Collection compound library was based on FCFP6 molecular fingerprints, or on nine Physicochemical Molecular Descriptors (PMD): AlogP (hydrophobicity), molecular weight, number of H donors, number of H acceptors, number of rotatable bonds, number of atoms, number of rings, number of aromatic rings and number of fragments. The FCFP6‐PCA and PMD‐PCA were performed using Pipeline Pilot software v9.2 (BIOVIA, San Diego, USA) and visualizations were made using JMP software v11.0.0 (SAS, Marlow, UK).

## 3 RESULTS

### 3.1 Screening of the Spectrum Collection bioactive compound library for SGLT1 inhibitors

A screening of 1956 compounds from the Spectrum Collection library for SGLT1 inhibitors resulted in identification of 108 primary hits and 1848 noninhibitors at an activity threshold of ≤70% of negative control, corresponding to a hit rate of 5.5%. A complete overview of screened compounds and their SGLT1 inhibitory activity can be found in Table S1.

### 3.2 Clusters of structurally similar molecules with SGLT1 and SGLT2 inhibitory activity

A selection of 30 hits from the primary SGLT1 inhibition screening and 101 additional structurally similar compounds were clustered using a Dice FCFP6 algorithm resulting in 20 clusters of one to 19 compounds with a mean intra‐cluster compound similarity of 74% ± 7%, excluding four single compound clusters. Both natural and synthetic compound clusters could be distinguished. For each compound cluster the inhibitory activity on SGLT1 and SGLT2 was determined at both 5 and 50 μmol/L. Clusters containing natural SGLT1/2 inhibiting compound(s) were: glycosides (14 compounds), angular pyranocoumarins (APCs) (19 compounds), quinidine‐like (5 compounds), polysaccharides (8 compounds) and viniferin‐like (10 compounds). Clusters containing synthetic SGLT1/2 inhibiting compounds were: trihexyphenidyl‐like (11 compounds), diphenhydramine‐like (11 compounds), bupivacaine‐like (13 compounds), trimipramine‐like (6 compounds), broxyquinoline‐like (5 compounds), perhexiline‐like (1 compound), C17H23N3O‐like (2‐[(3S)‐1‐(1‐methyl‐4‐piperidinyl)‐3‐pyrrolidinyl]‐1,3‐benzoxazole‐like) (4 compounds), C17H16FN3O2‐like (2‐[(3S)‐1‐(2‐fluorbenzyl)‐3‐pyrrolidinyl]‐5‐(2‐furyl)‐1,3,4‐oxadiazole‐like) (8 compounds) and triadimefon‐like (2 compounds). The intra‐cluster compound similarities, SGLT1 and SGLT2 inhibitory activities, and SGLT2/SGLT1 activity ratios to indicate selectivity are presented in Table S2.

**Figure 1:**
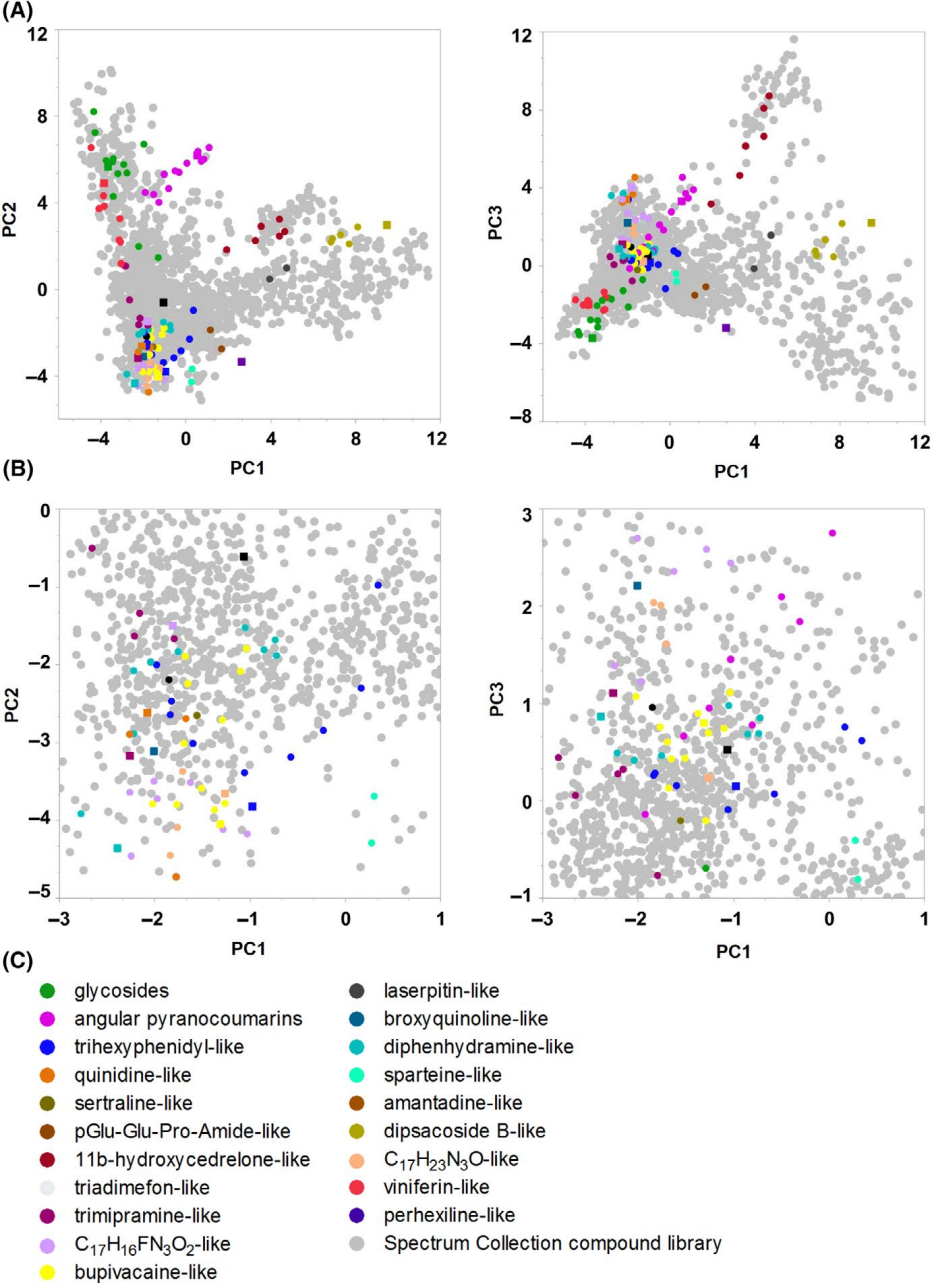
FCFP6‐PCA visualization of chemical space occupied by the Spectrum Collection compound library and chemical clusters with novel SGLT1/2 inhibitors. A, Chemical space visualization of the Spectrum Collection compound library and clusters of additional structurally similar molecules with PC1‐3 of the FCFP6‐PCA. Detected SGLT1/2 inhibitors in the clusters are indicated with ■, other cluster compounds with ●. SGLT1/2 inhibitors exist outside the chemical space of the phloridzin–containing glycosides cluster. B, Zoomed in image of A. C, Molecular clusters obtained with a Dice FCFP6 similarity algorithm. PC1‐3 of the FCFP6‐PCA explained 14% of intercompound differences. For the complete datasets of the PCA and Dice FCFP6 analyses, see Table S2

To visualize the separation of the different molecular clusters and the Spectrum Collection compounds in chemical space, PCAs were performed based on either the FCFP6 molecular fingerprints, or PMD. Principal Components (PC) 1‐3 of the FCFP6‐PCA together explained 14% (PC1 6.5%, PC2, 4.2%, PC3 3.5%) of the inter compound differences, while PC 1‐3 of the PMD‐PCA explained 77% (PC1 46%, PC2 19% and PC3 12%). Both PCAs showed that all molecular clusters were separated from the glycosides cluster, containing the canonical phloridzin and structurally related SGLT1/2 inhibitors. This shows that SGLT1 inhibitors exist outside the chemical space of glycosides. The separation of the clusters in chemical space was best visualized using the FCFP6‐PCA (Figure 1 and Figure S3).

**Figure 2:**
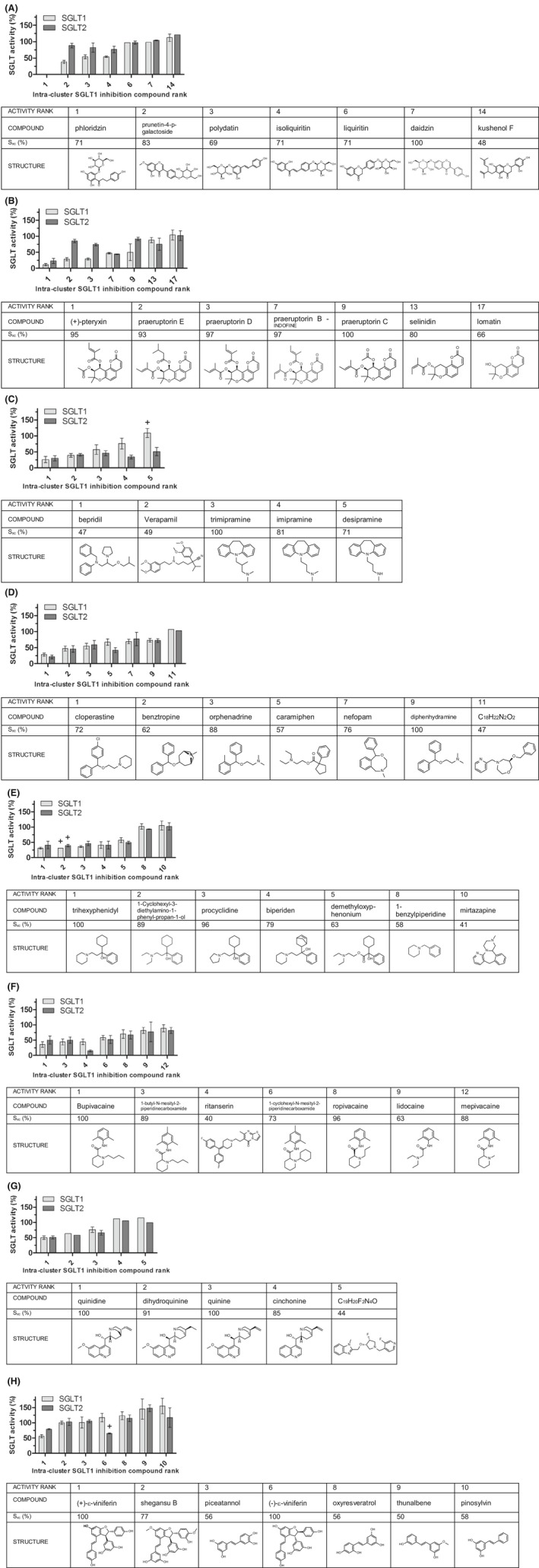
Compounds from different chemical clusters show varying inhibition of SGLT1 and SGLT2. Representative compounds per cluster are ranked from highest to lowest SGLT1 inhibitory activity at 50 μmol/L. For each compound x in a cluster the similarity to the central cluster compound c is indicated ( S_xc_(%)). Clusters in order of increasing activity are: A, glycosides (natural), (B) angular pyranocoumarins (APC, natural), (C) trimipramine‐like (synthetic), (D) diphenhydramine‐like (synthetic), (E), trihexyphenidyl‐like (synthetic), (F) bupivacaine‐like (synthetic), (G) quinidine‐like (natural), (H) viniferin‐like (natural). Results are means with SD from n biological replicates of two technical replicates, as indicated in Table S2. For means of n = 2 biological replicates SDs are used indicatively. + = original dataset for this compound contained a statistical outlier which was excluded from calculation of the mean and SD

Molecular features related to the SGLT1/2 inhibiting activity could be identified in some clusters. These and other features are described in the next paragraphs and shown in Figure 2. Unless stated otherwise, all results described are mean inhibition percentages obtained with 50 μmol/L inhibitor. Compounds with ≥30% inhibition were considered active inhibitors and clusters are described in order of increasing SGLT1 inhibition.

### 3.3 Glycosides

The canonical natural SGLT1/2 inhibitor phloridzin caused the strongest inhibition of all tested compounds on both SGLT1 and SGLT2 (Figure 2A, Table S2). The background 1‐NBD‐glucose uptake by SGLT1 and by SGLT2 with 50 μmol/L phloridzin was set at 0%, which equaled 100% inhibition. At 5 μmol/L, phloridzin still caused high nonselective inhibition of 91% of SGLT1 and 92% of SGLT2. Isoliquiritin, a natural dihydrochalcone glucoside like phloridzin showed 46% inhibition of SGLT1 and 24% inhibition of SGLT2 indicating some SGLT1 selectivity (SGLT2/SGLT1‐_50μmol/L_ of 1.4). Polydatin, a natural resveratrol glucoside, inhibited SGLT1 and SGLT2 with 46% and 18% respectively, also indicating a modest SGLT1 selectivity (SGLT2/SGLT1‐_50μmol/L_ of 1.5). Prunetin‐4‐p‐galactoside inhibited SGLT1 and SGLT2 with 62% and 12% respectively, demonstrating noteworthy SGLT1 selectivity (SGLT2/SGLT1‐_50μmol/L_ of 2.3).

### 3.4 Angular pyranocoumarins

The cluster of the natural APCs contained compounds that were second to phloridzin regarding SGLT1 inhibitory activity and showed SGLT1 selectivity. Substitution at the 9’ and 10’ C‐atom of the APC backbone with an alkyl or alkene ester of 2 to 5 C‐atoms was essential for SGLT1 inhibition, ranging from 89% for (+)‐pteryxin to 41% for one praeruptorin B (Figure 2B, Table S2). For one APC, the ester side chain at C‐atom 10’ contained a 2,3‐dimethyl‐2‐oxiranecarboxylate group which reduced SGLT1 inhibition to 27%. Selinidin, lacking the alkene substitution at the C‐10’, showed minimal inhibition of SGLT1 and SGLT2 (12% and 25% respectively). Lomatin and cis‐khellactone, which both lack alkene substitutions but have either a hydroxy‐group at C‐9’, or at C‐9’ and C‐10’ respectively, displayed no SGLT inhibition. Notably, for several of the SGLT1 inhibiting APCs substituted at C‐9’ and C‐10’ with alkyl or alkene ester side chains that inhibited SGLT1 with 50% to 72%, no to minimal inhibition of SGLT2 was observed indicating SGLT1 selectivity (respectively: praeruptorin C (SGLT2/SGLT1‐_50μmol/L_ of 1.8), peucedanocoumarin I (SGLT2/SGLT1‐_50μmol/L_ of 2), (+)‐praeruptorin A (SGLT2/SGLT1‐_50μmol/L_ of 2), praeruptorin A (SGLT2/SGLT1‐_50μmol/L_ of 3.1), praeruptorin D (SGLT2/SGLT1‐_50μmol/L_ of 2.6) and praeruptorin E (SGLT2/SGLT1‐_50μmol/L_ of 3.1). APCs that showed no inhibition of SGLT1 also did not show SGLT2 inhibition and none of the APCs showed selectivity for SGLT2.

### 3.5 Trimipramine–like compounds

In the trimipramine–like cluster, bepridil caused the strongest SGLT1 inhibition of 74% and SGLT2 inhibition of 70%, followed by verapamil (SGLT1 61%, SGLT2 59%) (Figure 2C, Table S2). These two compounds were the least similar to the cluster center, with S_xc_ 47% and 49% respectively. Results from a subcluster of most similar compounds suggested that SGLT1 inhibition is strongest with the *N,N*,2‐trimethyl‐1‐propanamine side chain on trimipramine (43% inhibition) and that inhibition is reduced when methyl groups are removed, as with imipramine (*N,N*‐dimethyl‐1‐propanamine side chain) (24% inhibition) and desipramine (*N*‐methyl‐1‐propanamine side chain) (no inhibition). These differences in activity were not observed for SGLT2 inhibition, indicating SGLT2 selectivity with an imipramine SGLT2/SGLT1‐_50μmol/L_ of 0.4 and a desipramine SGLT2/SGLT1‐_50μmol/L_ of 0.5.

### 3.6 Diphenhydramine–like compounds

Most (active) diphenhydramine–like compounds showed similar inhibition of SGLT1 (31%‐72%) or SGLT2 (34%‐79%). The inhibitory activity is related to two phenyl‐ring structures connected via a single C‐atom and an extending N–containing alkyl group attached via an O‐atom (Figure 2D, Table S2). Accordingly, cloperastine displayed the strongest SGLT1 inhibition of 72%. Caramiphen deviates structurally (S_xc_ of 57%) from the stronger SGLT1 inhibitors in this cluster by a substitution of one of the phenyl groups for a cyclopentyl group and attachment of the N–containing group via an ester bond instead of an O‐atom and, notably, showed stronger inhibition of SGLT2 (58%) than of SGLT1 (33%) (SGLT2/SGLT1‐_50μmol/L_ of 0.6).

### 3.7 Trihexyphenidyl–like compounds

Most active trihexyphenidyl–like compounds were good inhibitors of both SGLT1 (43%‐69%) and SGLT2 (40%‐61%) (Figure 2E, Table S2). Trihexyphenidyl, the central compound of this cluster, caused a considerable reduction in SGLT1 substrate uptake of 69%. For this cluster, a central 1‐phenyl‐propan‐1‐ol structure is important for SGLT inhibition, with the C‐1’ atom connected to an additional ring structure like cyclohexyl, or bicycloheptenyl, and the C‐3’ atom connected to an extending N–containing alkyl group, like pyrrolidinyl, piperidine, or diethylamine, as seen for the active diphenhydramine–like compounds. Compounds in this cluster with a lower S_xc_ showed lower SGLT1 inhibition. Minimal differences were observed between SGLT1 or SGLT2 inhibition.

### 3.8 Bupivacaine–like compounds

The SGLT1 inhibitory activity of the bupivacaine–like compounds is related to the presence of a piperidine group and the length of the attached N‐alkyl chain, with inhibitory activity of N‐butyl (bupivacaine, 64% inhibition)> N‐propyl (ropivacaine, 30% inhibition)> N‐methyl (mepivacaine, 11% inhibition) (Figure 2F, Table S2). No difference in inhibition was observed between a 2,6‐dimethylphenyl, or a 2,4,6‐trimethylphenyl group. Notably, unlike the other compounds in this cluster the structurally least similar compound ritanserin showed selectivity towards SGLT2 with an SGLT2/SGLT1‐_50μmol/L_ of 0.3.

### 3.9 Quinidine–like compounds

Quinidine–like compounds displayed inhibiting activity for both SGLT1 and SGLT2. Quinidine showed stronger SGLT1 inhibitory activity than its stereoisomer quinine, (50% and 24% respectively) (Figure 2G, Table S2). For SGLT2 inhibition, a smaller difference was observed between these stereoisomers (49% and 34% respectively). Dihydroquinine showed somewhat stronger inhibition than quinine.

### 3.10 Viniferin–like compounds

Isomers of the resveratrol dimer viniferin demonstrated differential SGLT1 or SGLT2 inhibition. (+)‐ε‐Viniferin showed considerable SGLT1 inhibition (44%) and little inhibition of SGLT2 (SGLT2/SGLT1‐_50μmol/L_ of 1.4). Conversely, its stereoisomer (−)‐ε‐viniferin did not inhibit SGLT1, but did show 35% inhibition of SGLT2 (SGLT2/SGLT1‐_50μmol/L_ of 0.6) (Figure 2H, Table S2). Thus, (+)‐ε‐viniferin and (−)‐ε‐viniferin show selectivity towards SGLT1 and SGLT2, respectively. Markedly, other tested stilbene polymers and monomers did not show any inhibitory activity except for Viniferol D that reduced substrate uptake by SGLT2 with 35%, but had no effect on SGLT1 (SGLT2/SGLT1‐_50μmol/L_ of 0.6).

### 3.11 SGLT1/2‐IC_50_‐values of identified novel inhibitors

The inhibitory activity of the most potent compound from relevant clusters was analyzed more accurately by determining IC_50_ values for 1‐NBD‐glucose uptake by SGLT1 and SGLT2 (Table 1). SGLT1/2‐IC_50_ values of found novel inhibitors were in the 10‐100 μmol/L range.

### 3.12 Caco‐2 SGLT1 inhibition by (+)‐pteryxin and Peucedanum praeruptorum extract

**Figure 3:**
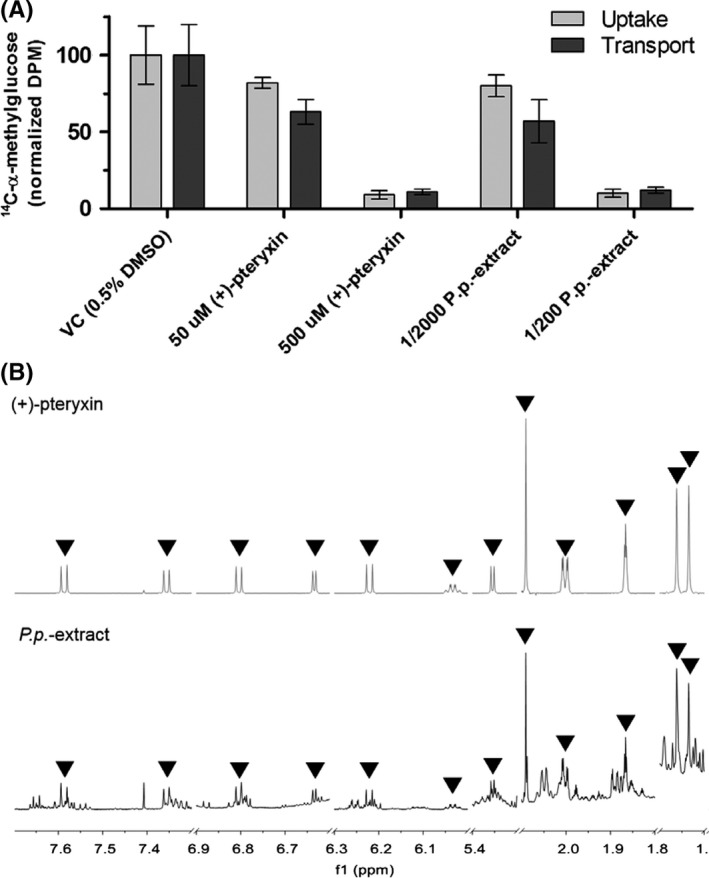
Inhibition of ^14^C‐α‐methylglucose uptake and transcellular transport by Caco‐2 cells by (+)‐pteryxin and *Peucedanum praeruptorum* extract containing (+)‐pteryxin. A, Inhibition of ^14^C‐α‐methylglucose cellular uptake and transcellular transport by (+)‐pteryxin and *P p*.‐extract. Results are means with SD from 2 biological replicates, each consisting of four technical replicates. VC = vehicle control. B, 700 MHz 1D ^1^H‐NMR spectra of (+)‐pteryxin (top) and *P p*.‐extract (bottom) dissolved in CDCl_3_ (298K). Characteristic signals from (+)‐pteryxin are annotated in both spectra using triangles, indicating the presence of (+)‐pteryxin in the *P p*.‐extract

The most potent identified novel natural inhibitor (+)‐pteryxin and an extract of the root of the plant *Peucedanum praeruptorum* (*P p*.‐extract), known to contain the APCs (+)‐pteryxin, peucedanocoumarin I and praeruptorins A to E,^36^ were tested for inhibition of uptake and transport of ^14^C‐α‐methylglucose by Caco‐2 cells expressing endogenous SGLT1. Qualitative NMR analyses confirmed the presence of (+)‐pteryxin in the *P p*.‐extract (Figure 3B). At 50 μmol/L and 500 μmol/L (+)‐pteryxin, ^14^C‐α‐methylglucose uptake was inhibited by 18% and 91% respectively, while the transcellular transport of ^14^C‐α‐methylglucose was inhibited by 37% and 89%. Dilutions of 1/2000 and 1/200 of the *P p*.‐extract inhibited cellular uptake of ^14^C‐α‐methylglucose by 20% and 90% respectively, while the transcellular transport was inhibited by 43% and 88% (Figure 3A).

## 4 DISCUSSION

We have tested natural and synthetic compounds for inhibition of the SLCs SGLT1/2. Novel inhibitors were identified in chemically different clusters that may serve as chemical leads for the treatment or prevention of T2DM. Analogs of the natural glycoside phloridzin are approved drugs that target renal SGLT2. In addition, intestinal SGLT1 is becoming recognized as a target for glycemic control.^7^, ^30^ Inhibition of SGLT1/2 by phloridzin is well established in vitro, but the hypoglycemic and glucosuric effects of phloridzin in vivo involve systemic and metabolic activities of its aglycon phloretin and further metabolites that are not entirely understood.^15^, ^16^ Hence, the primary aim of this study was to identify novel SGLT1 inhibitors outside the chemical space of phloridzin–like glycosides. Second, we aimed to produce a dataset of chemically diverse SGLT1/2 inhibitors for the development of in silico SGLT1 inhibitor screening models.^33^

A bioactive compound library covering a broad spectrum of chemical space was screened for SGLT1 inhibitors. Emerging from this screening, 131 compounds, both natural and synthetic, were further investigated for SGLT1/2 inhibition. Based on their molecular fingerprints, these compounds were grouped into 20 distinct chemical clusters. All clusters were separate from the cluster of glycosides. Some partially overlapped with each other when visualized with FCFP6‐PCA likely because of only minor structural differences between them. Moreover, using t–distributed stochastic neighbor embedding analysis, it was recently confirmed in a related study that the present SGLT1/2 inhibitor dataset is separated in chemical space from the public dataset in the compound database CheMBL.^33^, ^37^, ^38^ Thus, the current dataset expands the chemical diversity of SGLT1/2 inhibitors.

The SGLT1/2‐IC_50_‐values of the most potent novel inhibitors were in the 10‐100 μmol/L range. For some inhibitors, molecular features related to the inhibitory activity could be identified. To our knowledge, this study is the first to describe natural inhibitors showing (moderate) selectivity for SGLT1 over SGLT2. Cell viability analyses demonstrated that the inhibitory activities were not due to cytotoxicity artifacts (Table S2). The activity of the most potent novel natural inhibitor, (+)‐pteryxin, and a natural *P p*.‐extract containing this APC, was confirmed in a more physiologically relevant model using human intestinal SGLT1–expressing Caco‐2 cells with the glucose analog ^14^C‐α‐methylglucose as model substrate.

SGLT1–mediated transport of the glycoside polydatin, found in grape juice,^39^ has been reported.^40^ Polydatin ameliorated T2DM conditions in in vitro and animal models via various downstream mechanisms.^41^, ^42^, ^43^ Novel SGLT inhibiting glycosides found here are isoliquiritin from *Glycyrrhizae radix* (Liquorice) and prunetin‐4p‐galactoside from *Dalbergia spinose roxb*, which showed some SGLT1 selectivity. Another natural SGLT1 inhibitor identified is the resveratrol dimer (+)‐ε‐viniferin from the common grape V*itis vinifera*.^44^, ^45^ This confirms previous findings of ε‐viniferin reducing SGLT1 mediated glucose absorption in porcine jejunum and ileum.^46^ Results from the present study add that (+)‐ε‐viniferin shows moderate selectivity for SGLT1, while (−)‐ε‐viniferin displays modest but exclusive inhibition of SGLT2.

Other novel natural SGLT1/2 inhibitors identified are the dihydroxy‐APCs from *Peucedanum* plants. These plants have traditionally been indicated for treatment of obesity and hyperglycemia.^36^ Studies have described possible molecular mechanisms underlying the hypoglycemic effect of APCs or plant extracts containing these compounds,^47^, ^48^, ^49^, ^50^, ^51^, ^52^ but none of these studies revealed the SGLT1 inhibiting activity. Here, the presence of hydroxy‐ester groups on the 9’ and 10’ C‐atom of the APC backbone showed to be essential for SGLT inhibition. Notably, the praeruptorins A, D and E showed selectivity for SGLT1, while praeruptorin B did not.

Quinidine and quinine from cinchona tree bark are widely used antimalarials 53 that showed hitherto unreported SGLT1/2 inhibition. Notably, hypoglycemia is recognized as a comorbidity of malaria with complex multifactorial etiology, including quinine or quinidine induced hyperinsulinemia.^54^, ^55^, ^56^ Unchanged renal clearance has been shown for quinine,^57^ so it could be speculated that these drugs may inhibit intestinal SGLT1 and renal SGLT2, contributing to blood glucose lowering. To our knowledge, no studies have investigated glucosuria upon quinine and quinidine administration to support this hypothesis. Quinine is also used as a flavoring agent in beverages. It can be speculated that at the maximum FDA limit of 250 μmol/L per beverage, achievable gastrointestinal concentrations of about 200 μmol/L may partly inhibit SGLT1 (https://www.accessdata.fda.gov/scripts/cdrh/cfdocs/cfcfr/CFRSearch.cfm?fr=172.575). Overall, the above findings warrant further research in the application of the natural compounds isoliquiritin, prunetin‐4p‐galactoside, polydatin, (+)‐ε‐viniferin, APCs, quinine, or plant extracts containing these, as potential SGLT1 targeting treatments for glycemic control.

Identified novel SGLT1/2 inhibitors from the trihexyphenidyl–like cluster (trihexyphenidyl, procyclidine, biperiden) and the diphenhydramine–like cluster (cloperastine, benztropine, orphenadrine) are known anticholinergics and, except for cloperastine, M1 muscarinic receptor antagonists.^58^ Interestingly, anticholinergic drugs from other clusters, vesamicol and clemastine, also showed SGLT1/2 inhibition. Blood glucose lowering, or interactions with SGLT1/2 in individuals taking these drugs have not been investigated, but one case report showed reduced blood glucose in an oral glucose tolerance test with orphenadrine.^59^

The antianginal drug bepridil showed SGLT1/2 inhibition here as well. It was tested in T1DM and T2DM patients on antidiabetic medications, but no additional reduction in blood glucose levels was observed.^60^ The antiarrhythmic drug verapamil reduced the apparent Km for the (presumed) SGLT1–mediated uptake of D‐galactose by rabbit jejunum in vitro. Conversely, exposure of rabbit jejunal tissue to verapamil did not reduce D‐glucose uptake.^61^, ^62^ However, verapamil was shown to improve glucose tolerance 63 and reduce serum glucose levels in T2DM patients.^64^, ^65^ The local anesthetics bupivacaine, levobupivacaine and ropivacaine, as well as the antidepressant trimipramine also emerged as novel SGLT1/2 inhibitors in this study. Blood glucose lowering has been described for neither of them. Interestingly, many of the aforementioned approved drugs with novel identified SGLT1/2 inhibitory activity act via inhibition of various cation transporters including sodium linked cotransporters other than SGLT1/2 (Table S4).^58^

We applied a cellular screening assay to analyze the SGLT1/2 inhibitory activity of structurally diverse compounds using the fluorescent substrate 1‐NBD‐glucose instead of the radiolabelled close glucose analog ^14^C‐α‐methylglucose. This enabled the investigation of more than 2000 compounds using a standard microplate fluorescence reader while simultaneously limiting radioactive waste. Wu et al^66^ successfully applied a similar approach to screen for nonglycoside SGLT2 inhibitors and identified compounds with a benzimidazole scaffold as hits. 

The activity of the identified novel SGLT1 inhibitors with IC_50_‐values in the μmol/L‐range is modest compared to developed phloridzin–like SGLT1/2 inhibitors with nmol/L‐range IC_50_‐values. However, it can be speculated that depending on the dose and gastrointestinal stability, μmol/L‐range concentrations of these compounds may be achievable in the intestinal lumen at the intestinal wall SGLT1 target site after oral administration, before absorption and metabolism. For example, for the approved drugs with novel SGLT1/2 inhibition, efficacious gastrointestinal concentrations may be attainable at their highest prescribed single oral dose (Table S5). However, these drugs are designed to be rapidly absorbed and further study is required to determine whether effective concentrations are obtained and maintained at the intestinal SGLT1 target site.^58^ Possibly, applying a reversed pharmacokinetics optimization approach, these compounds provide a scaffold to develop nonabsorbable SGLT1 inhibitors analogous to the phloridzin–like inhibitor LX2761.^29^

Moreover, to enhance the potency and selectivity of the identified leads we suggest an active learning strategy based on PCM modeling.^33^, ^67^ This type of modeling incorporates data of both ligand and known protein targets without requiring 3D structural information making it particularly useful as a virtual screening model for transmembrane transporters like SGLT1/2. The PCM model is trained on existing data and used to virtually screen a library of novel compounds (or modified previously identified hits). Subsequently, a selection is made for experimental validation based on a high predicted activity but with a relatively modest probability.^68^ This is contrary to ordinary virtual screening wherein data points to be selected have a high predicted activity and probability. Hence, the model is used to identify data points that lead to the highest information gain (exploration) as opposed to identify newly active data points (exploitation). Previously this approach was shown to lead to a quick improvement in biological activity.^69^ Accordingly, using the current and a public dataset, a first PCM SGLT1 screening model was developed that effectively predicted moderately active SGLT1 inhibitors outside the chemical space of the training set.^33^ We expect further iterations of in silico and in vitro testing to improve this PCM SGLT1 model and the activity of its predicted hits.

In conclusion, we discovered novel natural and synthetic SGLT1/2 inhibitors beyond the chemical space of phloridzin and its analogs. The natural inhibitors are promising leads that may be further investigated as (prophylactic) agents to control dietary glucose uptake, for example as functional food ingredients or supplements. The synthetic inhibitors are mainly registered drugs indicated for conditions other than hyperglycemia. A blood glucose lowering (side) effect may be further investigated for these drugs. The new structure activity data from this study expands the existing public dataset to support further development of virtual SGLT1 inhibition screening models. Additional in vitro mechanistic studies are required to elucidate molecular interactions between the detected inhibitors and SGLT1/2. Finally, the new diverse structure activity data in this study provides starting points for development and optimization of novel, potent and selective SGLT1/2 inhibitors.

## CONFLICT OF INTERESTS

None.

## AUTHORS’ CONTRIBUTIONS

Participated in research design: Oranje, Gouka, Burggraaff, Vermeer, Chalet, Duchateau, van der Pijl, Geldof, Annaert, de Bruyn, Clauwaert, Nicolaï, Vanpaeschen, IJzerman, van Westen. Conducted experiments: Oranje, Gouka, Vermeer, de Roo, de Bruyn, Clauwaert, Nicolaï, Vanpaeschen. Contributed new reagents and analytic tools: Oranje, Gouka, Burggraaff, Vermeer, Chalet, van der Pijl, Annaert, IJzerman, van Westen. Performed data analysis: Oranje, Burggraaff, Vermeer, Chalet, de Roo. Contributed to writing of the manuscript: Oranje, Gouka, Burggraaff, Vermeer, Chalet, Duchateau, van der Pijl, Geldof, IJzerman, van Westen.

## References

[1] Zheng Y , Ley SH , Hu FB . Global aetiology and epidemiology of type 2 diabetes mellitus and its complications. Nat Rev Endocrinol. 2018;14(2):88‐98. 29219149 10.1038/nrendo.2017.151

[2] Madaan T , Akhtar M , Najmi AK . Sodium glucose CoTransporter 2 (SGLT2) inhibitors: Current status and future perspective. Eur J Pharm Sci. 2016;93:244‐252. 27531551 10.1016/j.ejps.2016.08.025

[3] Oyer DS . The science of hypoglycemia in patients with diabetes. Curr Diabetes Rev. 2013;9(3):195‐208. 23506375 10.2174/15733998113099990059

[4] Song P , Onishi A , Koepsell H , Vallon V . Sodium glucose cotransporter SGLT1 as a therapeutic target in diabetes mellitus. Expert Opin Ther Targets. 2016;20(9):1109‐1125. 26998950 10.1517/14728222.2016.1168808 PMC5045806

[5] Tahrani AA , Barnett AH , Bailey CJ . SGLT inhibitors in management of diabetes. Lancet Diabetes Endocrinol. 2013;1(2):140‐151. 24622320 10.1016/S2213-8587(13)70050-0

[6] Vallon V , Thomson SC . Targeting renal glucose reabsorption to treat hyperglycaemia: the pleiotropic effects of SGLT2 inhibition. Diabetologia. 2017;60(2):215‐225. 27878313 10.1007/s00125-016-4157-3 PMC5884445

[7] Spatola L , Finazzi S , Angelini C , Dauriz M , Badalamenti S . SGLT1 and SGLT1 inhibitors: a role to be assessed in the current clinical practice. Diabetes Ther. 2018;9(1):427‐430. 29177922 10.1007/s13300-017-0342-8 PMC5801228

[8] Wright EM , Loo DD , Hirayama BA . Biology of human sodium glucose transporters. Physiol Rev. 2011;91(2):733‐794. 21527736 10.1152/physrev.00055.2009

[9] Smith CD , Hirayama BA , Wright EM . Baculovirus‐mediated expression of the Na+/glucose cotransporter in Sf9 cells. Biochem Biophys Acta. 1992;1104(1):151‐159. 1550844 10.1016/0005-2736(92)90144-b

[10] Kanai Y , Lee WS , You G , Brown D , Hediger MA . The human kidney low affinity Na+/glucose cotransporter SGLT2. Delineation of the major renal reabsorptive mechanism for D‐glucose. J Clin Investig. 1994;93(1):397‐404. 8282810 10.1172/JCI116972 PMC293794

[11] Hummel CS , Lu C , Liu J , et al. Structural selectivity of human SGLT inhibitors. Am J Physiol Cell Physiol. 2012;302(2):C373‐C382. 21940664 10.1152/ajpcell.00328.2011 PMC3328840

[12] Von Mering J . Über künstlichen diabetes. Centralbl Med Wiss. 1886;22:531.

[13] Rossetti L , Smith D , Shulman GI , Papachristou D , DeFronzo RA . Correction of hyperglycemia with phlorizin normalizes tissue sensitivity to insulin in diabetic rats. J Clin Investig. 1987;79(5):1510‐1515. 3571496 10.1172/JCI112981 PMC424427

[14] Katsuda Y , Sasase T , Tadaki H , et al. Contribution of hyperglycemia on diabetic complications in obese type 2 diabetic SDT fatty rats: effects of SGLT inhibitor phlorizin. Exp Anim. 2015;64(2):161‐169. 25736710 10.1538/expanim.14-0084 PMC4427731

[15] Schulze C , Bangert A , Kottra G , et al. Inhibition of the intestinal sodium‐coupled glucose transporter 1 (SGLT1) by extracts and polyphenols from apple reduces postprandial blood glucose levels in mice and humans. Mol Nutr Food Res. 2014;58(9):1795‐1808. 25074384 10.1002/mnfr.201400016

[16] Makarova E , Gornas P , Konrade I , et al. Acute anti‐hyperglycaemic effects of an unripe apple preparation containing phlorizin in healthy volunteers: a preliminary study. J Sci Food Agric. 2015;95(3):560‐568. 24917557 10.1002/jsfa.6779

[17] Ehrenkranz JR , Lewis NG , Kahn CR , Roth J . Phlorizin: a review. Diabetes Metab Res Rev. 2005;21(1):31‐38. 15624123 10.1002/dmrr.532

[18] Meng W , Ellsworth BA , Nirschl AA , et al. Discovery of dapagliflozin: a potent, selective renal sodium‐dependent glucose cotransporter 2 (SGLT2) inhibitor for the treatment of type 2 diabetes. J Med Chem. 2008;51(5):1145‐1149. 18260618 10.1021/jm701272q

[19] Nomura S , Sakamaki S , Hongu M , et al. Discovery of canagliflozin, a novel C‐glucoside with thiophene ring, as sodium‐dependent glucose cotransporter 2 inhibitor for the treatment of type 2 diabetes mellitus. J Med Chem. 2010;53(17):6355‐6360. 20690635 10.1021/jm100332n

[20] Grempler R , Thomas L , Eckhardt M , et al. Empagliflozin, a novel selective sodium glucose cotransporter‐2 (SGLT‐2) inhibitor: characterisation and comparison with other SGLT‐2 inhibitors. Diabetes Obes Metab. 2012;14(1):83‐90. 21985634 10.1111/j.1463-1326.2011.01517.x

[21] Zhang XL , Zhu QQ , Chen YH , et al. Cardiovascular safety, long‐term noncardiovascular safety, and efficacy of sodium‐glucose cotransporter 2 inhibitors in patients with type 2 diabetes mellitus: a systemic review and meta‐analysis with trial sequential analysis. J Am Heart Assoc. 2018;7(2). 10.1161/JAHA.117.007165 PMC5850151 29353233

[22] Yale JF , Bakris G , Cariou B , et al. Efficacy and safety of canagliflozin in subjects with type 2 diabetes and chronic kidney disease. Diabetes Obes Metab. 2013;15(5):463‐473. 23464594 10.1111/dom.12090 PMC3654568

[23] Kuo GH , Gaul MD , Liang Y , et al. Synthesis and biological evaluation of benzocyclobutane‐C‐glycosides as potent and orally active SGLT1/SGLT2 dual inhibitors. Bioorg Med Chem Lett. 2018;28(7):1182‐1187. 29523385 10.1016/j.bmcl.2018.02.057

[24] Tsimihodimos V , Filippas‐Ntekouan S , Elisaf M . SGLT1 inhibition: Pros and cons. Eur J Pharmacol. 2018;838:153‐156. 30240793 10.1016/j.ejphar.2018.09.019

[25] Rosenstock J , Cefalu WT , Lapuerta P , et al. Greater dose‐ranging effects on A1C levels than on glucosuria with LX4211, a dual inhibitor of SGLT1 and SGLT2, in patients with type 2 diabetes on metformin monotherapy. Diabetes Care. 2015;38(3):431‐438. 25216510 10.2337/dc14-0890 PMC5131876

[26] Zambrowicz B , Ogbaa I , Frazier K , et al. Effects of LX4211, a dual sodium‐dependent glucose cotransporters 1 and 2 inhibitor, on postprandial glucose, insulin, glucagon‐like peptide 1, and peptide tyrosine tyrosine in a dose‐timing study in healthy subjects. Clin Ther. 2013;35(8):1162‐1173.e1168. 23911260 10.1016/j.clinthera.2013.06.011

[27] Lapuerta P , Rosenstock J , Zambrowicz B , et al. Study design and rationale of a dose‐ranging trial of LX4211, a dual inhibitor of SGLT1 and SGLT2, in type 2 diabetes inadequately controlled on metformin monotherapy. Clin Cardiol. 2013;36(7):367‐371. 23630033 10.1002/clc.22125 PMC6649411

[28] Powell DR , Smith M , Greer J , et al. LX4211 increases serum glucagon‐like peptide 1 and peptide YY levels by reducing sodium/glucose cotransporter 1 (SGLT1)‐mediated absorption of intestinal glucose. J Pharmacol Exp Ther. 2013;345(2):250‐259. 23487174 10.1124/jpet.113.203364

[29] Goodwin NC , Ding ZM , Harrison BA , et al. Discovery of LX2761, a sodium‐dependent glucose cotransporter 1 (SGLT1) inhibitor restricted to the intestinal lumen, for the treatment of diabetes. J Med Chem. 2017;60(2):710‐721. 28045524 10.1021/acs.jmedchem.6b01541

[30] Powell DR , Smith MG , Doree DD , et al. LX2761, a sodium/glucose cotransporter 1 inhibitor restricted to the intestine, improves glycemic control in mice. J Pharmacol Exp Ther. 2017;362(1):85‐97. 28442582 10.1124/jpet.117.240820

[31] Lehmann A , Hornby PJ . Intestinal SGLT1 in metabolic health and disease. Am J Physiol Gastrointest Liver Physiol. 2016;310(11):G887‐898. 27012770 10.1152/ajpgi.00068.2016

[32] Chang HC , Yang SF , Huang CC , et al. Development of a novel non‐radioactive cell‐based method for the screening of SGLT1 and SGLT2 inhibitors using 1‐NBDG. Mol BioSyst. 2013;9(8):2010‐2020. 23657801 10.1039/c3mb70060g

[33] Burggraaff L , Oranje P , Gouka R , et al. Identification of novel small molecule inhibitors for solute carrier SGLT1 using proteochemometric modeling. J Cheminform. 2019;11(1):15. 30767155 10.1186/s13321-019-0337-8 PMC6689890

[34] Steffansen B , Pedersen M , Laghmoch AM , Nielsen CU . SGLT1‐mediated transport in Caco‐2 cells is highly dependent on cell bank origin. J Pharm Sci. 2017;106(9):2664‐2670. 28454747 10.1016/j.xphs.2017.04.033

[35] Dice LR . Measures of the amount of ecologic association between species. Ecology. 1945;26(3):297‐302.

[36] Sarkhail P . Traditional uses, phytochemistry and pharmacological properties of the genus Peucedanum: a review. J Ethnopharmacol. 2014;156:235‐270. 25193684 10.1016/j.jep.2014.08.034

[37] Gaulton A , Bellis LJ , Bento AP , et al. ChEMBL: a large‐scale bioactivity database for drug discovery. Nucleic Acids Res. 2012;40(Database issue):D1100‐D1107. 21948594 10.1093/nar/gkr777 PMC3245175

[38] Gaulton A , Hersey A , Nowotka M , et al. The ChEMBL database in 2017. Nucleic Acids Res. 2017;45(D1):D945‐D954. 27899562 10.1093/nar/gkw1074 PMC5210557

[39] Romero‐Perez AI , Ibern‐Gomez M , Lamuela‐Raventos RM , de La Torre‐Boronat MC . Piceid, the major resveratrol derivative in grape juices. J Agric Food Chem. 1999;47(4):1533‐1536. 10564012 10.1021/jf981024g

[40] Henry C , Vitrac X , Decendit A , Ennamany R , Krisa S , Merillon JM . Cellular uptake and efflux of trans‐piceid and its aglycone trans‐resveratrol on the apical membrane of human intestinal Caco‐2 cells. J Agric Food Chem. 2005;53(3):798‐803. 15686436 10.1021/jf048909e

[41] Wang Y , Ye J , Li J , et al. Polydatin ameliorates lipid and glucose metabolism in type 2 diabetes mellitus by downregulating proprotein convertase subtilisin/kexin type 9 (PCSK9). Cardiovasc Diabetol. 2016;15:19. 26833058 10.1186/s12933-015-0325-x PMC4736185

[42] Zhang Q , Tan Y , Zhang N , Yao F . Polydatin supplementation ameliorates diet‐induced development of insulin resistance and hepatic steatosis in rats. Mol Med Rep. 2015;11(1):603‐610. 25333896 10.3892/mmr.2014.2708

[43] Hao J , Chen C , Huang K , et al. Polydatin improves glucose and lipid metabolism in experimental diabetes through activating the Akt signaling pathway. Eur J Pharmacol. 2014;745:152‐165. 25310908 10.1016/j.ejphar.2014.09.047

[44] Douillet‐Breuil AC , Jeandet P , Adrian M , Bessis R . Changes in the phytoalexin content of various Vitis spp. in response to ultraviolet C elicitation. J Agric Food Chem. 1999;47(10):4456‐4461. 10552833 10.1021/jf9900478

[45] Ohara K , Kusano K , Kitao S , Yanai T , Takata R , Kanauchi O . epsilon‐Viniferin, a resveratrol dimer, prevents diet‐induced obesity in mice. Biochem Biophys Res Comm. 2015;468(4):877‐882. 26596701 10.1016/j.bbrc.2015.11.047

[46] Guschlbauer M , Klinger S , Burmester M , Horn J , Kulling SE , Breves G . trans‐Resveratrol and epsilon‐viniferin decrease glucose absorption in porcine jejunum and ileum in vitro. Comp Biochem Physiol A Mol Integr Physiol. 2013;165(3):313‐318. 23570675 10.1016/j.cbpa.2013.03.040

[47] Nugara RN , Inafuku M , Iwasaki H , Oku H . Partially purified Peucedanum japonicum Thunb extracts exert anti‐obesity effects in vitro. Nutrition. 2014;30(5):575‐583. 24698349 10.1016/j.nut.2013.09.017

[48] Nugara RN , Inafuku M , Takara K , Iwasaki H , Oku H . Pteryxin: a coumarin in Peucedanum japonicum Thunb leaves exerts antiobesity activity through modulation of adipogenic gene network. Nutrition. 2014;30(10):1177‐1184. 24993752 10.1016/j.nut.2014.01.015

[49] Kumar A , Maurya RA , Sharma S , et al. Pyranocoumarins: a new class of anti‐hyperglycemic and anti‐dyslipidemic agents. Bioorg Med Chem Lett. 2009;19(22):6447‐6451. 19811915 10.1016/j.bmcl.2009.09.031

[50] Nukitrangsan N , Okabe T , Toda T , Inafuku M , Iwasaki H , Oku H . Effect of Peucedanum japonicum Thunb extract on high‐fat diet‐induced obesity and gene expression in mice. J Oleo Sci. 2012;61(2):89‐101. 22277893 10.5650/jos.61.89

[51] Okabe T , Toda T , Nukitrangsan N , Inafuku M , Iwasaki H , Oku H . Peucedanum japonicum Thunb inhibits high‐fat diet induced obesity in mice. Phytother Res. 2011;25(6):870‐877. 21108487 10.1002/ptr.3355

[52] Choi RY , Nam SJ , Ham JR , et al. Anti‐adipogenic and anti‐diabetic effects of cis‐3',4'‐diisovalerylkhellactone isolated from Peucedanum japonicum Thunb leaves in vitro. Bioorg Med Chem Lett. 2016;26(19):4655‐4660. 27575482 10.1016/j.bmcl.2016.08.056

[53] Cechinel‐Filho V . Plant Bioactives and Drug Discovery: Principles, Practice, and Perspectives. Hoboken, N.J: John Wiley & Sons; 2012.

[54] Roe JK , Pasvol G . New developments in the management of malaria in adults. QJM. 2009;102(10):685‐693. 19656846 10.1093/qjmed/hcp087

[55] Davis TM , Pukrittayakamee S , Supanaranond W , et al. Glucose metabolism in quinine‐treated patients with uncomplicated falciparum malaria. Clin Endocrinol. 1990;33(6):739‐749. 10.1111/j.1365-2265.1990.tb03911.x 2096009

[56] Thien HV , Kager PA , Sauerwein HP . Hypoglycemia in falciparum malaria: is fasting an unrecognized and insufficiently emphasized risk factor? Trends Parasitol. 2006;22(9):410‐415. 16839817 10.1016/j.pt.2006.06.014

[57] Wanwimolruk S , Chalcroft S , Coville PF , Campbell AJ . Pharmacokinetics of quinine in young and elderly subjects. Trans R Soc Trop Med Hyg. 1991;85(6):714‐717. 1801332 10.1016/0035-9203(91)90423-v

[58] Wishart DS , Feunang YD , Guo AC , et al. DrugBank 5.0: a major update to the DrugBank database for 2018. Nucleic Acids Res. 2018;D1074‐d1082. 29126136 10.1093/nar/gkx1037 PMC5753335

[59] Buckle RM . Hypoglycaemic coma occurring during treatment with chlorpromazine and orphenadrine. BMJ. 1967;4(5579):599‐600. 6060121 10.1136/bmj.4.5579.599 PMC1749255

[60] Husted SE , Pedersen C , Nielsen HK , et al. Effect of bepridil on metabolic control and insulin secretion in diabetics. Eur J Clin Pharmacol. 1988;34(3):221‐226. 3294020 10.1007/BF00540947

[61] Hyson DA , Thomson AB , Kappagoda CT . Calcium channel blockers modify jejunal uptake of D‐galactose in rabbits. Dig Dis Sci. 1996;41(9):1871‐1875. 8794809 10.1007/BF02088760

[62] Hyson DH , Thomson AB , Keelan M , Kappagoda CT . A high cholesterol diet blocks the effect of calcium channel blockers on the uptake of sugars in rabbit intestine. Can J Physiol Pharmacol. 1997;75(1):57‐64. 9101066

[63] Andersson DE , Rojdmark S . Improvement of glucose tolerance by verapamil in patients with non‐insulin‐dependent diabetes mellitus. Acta medica Scandinavica. 1981;210(1–2):27‐33. 7027745 10.1111/j.0954-6820.1981.tb09771.x

[64] Khodneva Y , Shalev A , Frank SJ , Carson AP , Safford MM . Calcium channel blocker use is associated with lower fasting serum glucose among adults with diabetes from the REGARDS study. Diabetes Res Clin Pract. 2016;115:115‐121. 26818894 10.1016/j.diabres.2016.01.021 PMC4887408

[65] Yin T , Kuo SC , Chang YY , Chen YT , Wang KK . Verapamil use is associated with reduction of newly diagnosed diabetes mellitus. J Clin Endocrinol Metab. 2017;102(7):2604‐2610. 28368479 10.1210/jc.2016-3778

[66] Wu SH , Yao CH , Hsieh CJ , et al. Development and application of a fluorescent glucose uptake assay for the high‐throughput screening of non‐glycoside SGLT2 inhibitors. Eur J Pharm Sci. 2015;74:40‐44. 25819489 10.1016/j.ejps.2015.03.011

[67] van Westen G , Wegner JK , Ijzerman AP , van Vlijmen H , Bender A . Proteochemometric modeling as a tool to design selective compounds and for extrapolating to novel targets. MedChemComm. 2011;2(1):16‐30.

[68] Reker D , Schneider P , Schneider G , Brown JB . Active learning for computational chemogenomics. Future Med Chem. 2017;9(4):381‐402. 28263088 10.4155/fmc-2016-0197

[69] Cortes‐Ciriano I , Firth NC , Bender A , Watson O . Discovering highly potent molecules from an initial set of inactives using iterative screening. J Chem Inf Model. 2018;58(9):2000‐2014. 30130102 10.1021/acs.jcim.8b00376

